# Investigation of functional constipation in elderly inpatients and analysis of its influencing factors: A cross-sectional study

**DOI:** 10.1097/MD.0000000000039624

**Published:** 2024-09-06

**Authors:** Xuejiao Xian, Xiaobin Wang, Jie Liu, Hongjun Yang

**Affiliations:** a Department of General Surgery, Chuxiong Yi Autonomous Prefecture People’s Hospital, Chuxiong City, Yunnan Province, China; b Department of Geriatric General Surgery, The First Affiliated Hospital of Kunming Medical University, Kunming, Yunnan, China.

**Keywords:** elderly, functional constipation, influencing factors, investigation

## Abstract

To investigate the prevalence of functional constipation (FC) in elderly hospitalized patients and analyze the influencing factors. This was a cross-sectional study in which 506 patients hospitalized in general surgery from February to June 2020 were selected. Information on patients’ age, gender, ethnicity, body mass index, intake of vegetables, fruits, meat, and spicy foods, sleep, smoking, alcohol consumption, time of defecation, and mode of defecation was collected through questionnaires, and the factors affecting functional constipation were analyzed using binary logistic regression models; among 506 patients, 254 had FC, with a prevalence of 50.19%. Among the clinical symptoms of FC, the most common ones were straining to defecate (83.85%) and lumpy or hard stools (81.80%). Univariate analysis revealed statistically significant differences in family history of constipation (*P* = .033), sedentary (*P* = .004), self-care ability (*P* = .001), body mass index (*P* = .013), defecation time (*P* < .0001), spicy food intake in dietary preference (*P* = .001), age (*P* = .004), and education level (*P* = .016), and binary logistic regression analysis showed that defecation time and spicy food consumption were independent influencing factors of FC. For hospitalized elderly people, regular morning defecation and not eating spicy foods can more helpful to slow the occurrence of functional constipation.

## 1. Introduction

Functional constipation (FC) is a common functional gastrointestinal disorder, the main clinical manifestations of which are reduced frequency of defecation, difficult or incomplete defecation, excluding organic pathology, and does not meet the diagnostic criteria of irritable bowel syndrome.^[1]^ With the development of society, the aging of the population is becoming more and more prominent, and the dietary structure has changed greatly, the incidence of FC has increased significantly. The pooled overall prevalence of FC using the Rome criteria was 8.5% in China.^[1]^ Between 1991 and 2020, the overall prevalence of FC showed a steadily increasing trend, reaching a maximum of 10.9%. According to literature, the prevalence of FC in China ranges from 3.0% to 17.6%,^[2]^ and the prevalence of FC in elderly people aged 60 years and above ranges from 15% to 20%,^[3]^ and the prevalence of FC in elderly people aged 80 years and above can be as high as 38%.^[4]^ The prevalence of FC in elderly women is higher, 2 to 3 times that of elderly men.^[5]^ The prevalence of FC in elderly inpatients is also as high as 33.5%.^[6]^ There is a lack of research data on this group in Yunnan. Therefore, the aim of this study was to investigate the prevalence of FC in elderly hospitalized patients and analyze the possible influencing factors to help clinical diagnosis and treatment.

## 2. Information and methods

### 2.1. General information

Patients hospitalized in the First Affiliated Hospital of Kunming Medical University and Chuxiong Yi Autonomous Prefecture People’s Hospital from February to June 2020 were used for the survey, and the diagnosis of FC was made according to the Rome IV criteria. Exclusion criteria: (1) age < 60 years; (2) incomplete information in response; (3) difficulty in verbal communication; (4) combined gastrointestinal malignancy and irritable bowel syndrome; (5) cognitive dysfunction.

We recruited a total of 600 patients, of whom 63 were excluded because of incomplete questionnaire information, 24 were excluded because of comorbid gastrointestinal tumors, 7 were excluded because they refused to continue participating in the questionnaire midway through the process. A total of 506 cases were included in this study, including 281 males and 225 females, aged 60 to 96 years, with a median age of 74 years.

These 506 patients were hospitalized for cardiovascular and cerebrovascular diseases, pulmonary diseases, inguinal hernia, thyroid diseases, and other non-gastrointestinal diseases.

### 2.2. Methods

The questionnaire for this study was designed with reference to the Rome IV standard. The questionnaire included the demographic data of the study subjects: age, gender, ethnicity, education, place of residence (urban/rural), body mass index (BMI) (undernourished/normal/overweight/obese); lifestyle habits: consumption of vegetables, fruits, meat, and spicy foods (1 degree for hardly eating/ 2 degrees for eating 1–2 times a week/ 3 degrees for eating 3–4 times a week/ 4 degrees for eating 4–5 times a week/ 4 degrees for eating every day eat as 5 degrees), water consumption (<700 mL/700–1500 mL/>1500 mL per day), daily activity (<30 min/≥30 min), sleep (often staying up late/not staying up late), self-care ability (fully self-care/partially self-care/not self-care), sedentary, smoking, alcohol consumption, family history of constipation; mental psychological factors: life stress, mental status (depression/ anxiety/ normal); defecation status: time of defecation (morning/ midday/ evening/ irregular), defecation method (squatting pit/ toilet), constipation symptoms, etc. The data were recorded in detail after completing the questionnaire for subsequent statistical analysis.

### 2.3. Diagnostic criteria

Using the Rome IV standard, the symptoms of FC must include two or more of the following: (a) Straining more than 25% of defecations. (b) Lumpy or hard stools more than 25% of defecations. (c) Sensation of incomplete evacuation more than one-fourth (25%) of defecations. (d) Sensation of anorectal obstruction/blockage more than one-fourth (25%) of defecations. (e) Manual maneuvers to facilitate more than one-fourth (25%) of defecations. (f) Fewer than three spontaneous bowel movements per week.^[1]^

### 2.4. Statistical analysis

Data were processed using SPSS 17.0 software. Count data were expressed by chi-square test, measurement data were expressed by mean ± standard deviation ($\bar{\chi }\pm s$), *t* test was used for comparison between groups, and multi-factor analysis was performed by multi-factor logistic regression analysis, and *P* < .05 was considered statistically significant difference.

## 3. Results

### 3.1. FC in elderly inpatients in Yunnan

Two hundred fifty-four out of 506 elderly inpatients (50.19%) had FC. Among the clinical symptoms of FC, the most common ones were straining to defecate (83.85%) and lumpy or hard stool (81.80%), see Table 1; 96 (37.8%) had 2 symptoms at the same time, 158 (62.2%) had 3 or more symptoms. (62.2%), of which 4 people (1.57%) had all 6 symptoms, see Table 2 for details.

**Table 1:** Proportion of constipation symptoms in elderly inpatients with FC in Kunming area.

Symptoms	Male (%)	Female (%)	Total
Straining to defecate	124 (89.85)	89 (76.72)	213 (83.85)
Lumpy or hard stools	115 (83.33)	91 (78.44)	206 (81.10)
Feeling of incomplete bowel movement	78 (56.52)	75 (64.65)	153 (60.24)
Feeling of anorectal obstruction	43 (31.15)	40 (34.48)	83 (32.67)
Need to use hands to promote bowel movement	24 (17.39)	13 (11.20)	37 (14.57)
Defecation <3 times a week	43 (31.15)	45 (38.79)	88 (34.65)

**Table 2:** Information table of symptoms of constipation in elderly inpatients with FC.

Presence of symptoms	Percentage	Number of people
2 symptoms	96	37.80
3 types of symptoms	72	28.35
4 symptoms	60	23.62
5 symptoms	22	8.66
6 symptoms	4	1.57
Total	254	100.0

### 3.2. Univariate analysis of FC in elderly inpatients in Yunnan

There were no statistically significant differences in ethnicity, gender, smoking, alcohol consumption, sleep, place of residence, exercise, life stress, defecation pattern, water intake, psychosocial status, and intake of meat, vegetables, and fruits in dietary preference (*P* > .05); there were statistically significant differences in family history of constipation, sedentary, self-care ability, BMI, time of defecation, intake of spicy foods in dietary preference, age, and education (all *P* < .05). There were statistically significant differences in family history of constipation, sedentary, self-care ability, BMI, defecation time, intake of spicy foods in dietary preference, age, and education level (all *P* < .05), as shown in Table 3.

**Table 3:** Single factor analysis of count data of FC in elderly inpatients in Kunming area (case).

Variables	Non-FC group (n = 252)	FC Group (n = 254)	Statistical quantities	*P*-value
Age	73.61 ± 9.12	75.97 ± 9.05	−2.929	.004
Ethnicity			0.066	.797
Han Chinese	226 (89.68%)	226 (88.98%)		
Ethnic Minorities	26 (10.32%)	28 (11.02%)		
Gender			0.299	.585
Male	143 (56.75%)	138 (54.33%)		
Female	109 (43.25%)	116 (45.47%)		
Family history of constipation			4.566	.033
None	236 (93.65%)	224 (88.19%)		
There are	16 (6.35%)	30 (11.81%)		
Smoking			2.135	.144
No	192 (76.19%)	207 (81.50%)		
Yes	60 (23.81%)	47 (18.50%)		
Drinking			3.564	.059
No	218 (86.51%)	233 (91.73%)		
Yes	34 (13.49%)	21 (8.27%)		
Sleep			0.591	.442
No late nights	223 (88.49%)	219 (86.22%)		
Frequent late nights	29 (11.51%)	35 (13.78%)		
Education level			5783	.016
College or above	126 (50.00%)	154 (60.63%)		
Under college	126 (50.00%)	100 (39.37%)		
Urban or rural			0.929	.335
Rural	50 (19.84%)	42 (16.54%)		
City	202 (80.16%)	212 (83.46%)		
Amount of exercise			3.535	.060
<30 minutes	47 (18.65%)	65 (25.60%)		
≥30 minutes	205 (81.35%)	189 (74.40%)		
Life is stressful			0.265	.606
No	230 (91.27%)	235 (91.52%)		
Yes	22 (8.73%)	19 (7.48%)		
Prolonged sitting			8.128	.004
No	191 (75.79%)	163 (64.17%)		
Yes	61 (24.21%)	91 (35.83%)		
Defecation method			0.349	.555
Squatting pit	56 (22.22%)	51 (20.08%)		
Toilet	196 (77.78%)	203 (79.92%)		
Water consumption			4.104	.128
≤700 mL	45 (17.86%)	55 (21.65%)		
700–1500 mL	118 (46.82%)	130 (51.18%)		
≥1500 mL	89 (35.32%)	69 (27.17%)		
Spiritual psychological condition			0.727	.692
Anxiety	35 (13.89%)	30 (11.81%)		
Depression	2 (0.79%)	3 (1.18%)		
Normal	215 (85.32%)	221 (87.01%)		
Self-care ability			13.350	.001
Cannot take care of themselves	4 (1.59%)	10 (3.94%)		
Partially self-care	66 (26.19%)	99 (38.98%)		
Fully self-care	182 (72.22%)	145 (57.08%)		
BMI			10.832	.013
Malnutrition	21 (8.33%)	39 (15.35%)		
Normal	142 (56.35%)	150 (59.06%)		
Overweight	65 (25.79%)	53 (20.87%)		
Obesity	24 (9.53%)	12 (4.72%)		
Defecation time			25.887	<.0001
unscheduled	92 (36.51%)	148 (58.27%)		
Morning	148 (58.73%)	96 (37.80%)		
Medium	2 (0.79%)	4 (1.57%)		
Evening	10 (3.97%)	6 (2.36%)		
Likes to eat vegetables			8.197	.073
1	2 (0.79%)	2 (0.80%)		
2	3 (1.19%)	12 (4.72%)		
3	31 (12.30%)	36 (14.17%)		
4	35 (13.89%)	44 (17.32%)		
5	181 (71.83%)	160 (62.99%)		
Like to eat fruits			5.465	.243
1	27 (10.71%)	43 (16.93%)		
2	70 (27.78%)	58 (22.83%)		
3	36 (14.28%)	41 (16.14%)		
4	9 (3.58%)	7 (2.76%)		
5	110 (43.65%)	105 (41.34%)		
Spicy diet			18.675	.001
1	155 (61.51%)	171 (67.32%)		
2	33 (13.10%)	51 (20.08%)		
3	13 (5.16%)	4 (1.57%)		
4	1 (0.39%)	3 (1.18%)		
5	50 (19.84%)	25 (9.85%)		
Likes to eat meat			7.216	.125
1	28 (11.11%)	36 (14.17%)		
2	39 (15.48%)	51 (20.08%)		
3	24 (9.53%)	33 (12.99%)		
4	8 (3.17%)	4 (1.58%)		
5	153 (60.71%)	130 (51.18%)		

### 3.3. Binary logistic regression analysis of factors influencing FC in elderly inpatients in Yunnan

Covariate diagnoses of family history of constipation, age, literacy, sedentary, ability to take care of oneself, BMI, time to defecation, and spicy food intake had VIFs <10, so logistic analysis could be performed. A binary logistic regression analysis was performed with the occurrence of FC as the dependent variable (no = 0, yes = 1) and the factors with *P* < .05 in the univariate analysis including family history of constipation, education, sedentary, self-care ability, BMI, defecation time, and spicy food intake as independent variables. It was further concluded that *P* < .05 for time of defecation and spicy food intake were independent influencing factors for the occurrence of FC. The rest of sedentary, BMI, literacy, self-care ability, and family history of constipation were not independent influencing factors for the occurrence of FC. In the time of defecation, the occurrence of FC with regular defecation in the morning was 42.7% of that with irregular defecation; in the influencing factor of spicy food intake, the occurrence of FC with 2 degrees of spicy food intake (1–2 times a week) was 1.827 times of that with 1 degree (no spicy food). This suggests that the prevalence of FC is lower with regular morning bowel movements and no spicy food (see Table 4).

**Table 4:** Logistic regression analysis of influencing factors of FC in elderly hospitalized patients in Kunming area.

		B	S.E.	Wals	df	Sig.	Exp (B)	95% of EXP (B) C.I.	
								Lower limit	Upper limit
Step 1*	Age	.004	.012	.118	1	.731	1.004	.981	1.028
	Family history of constipation (1)	.563	.341	2.723	1	.099	1.755	.900	3.425
	Literacy (1)	.393	.201	3.804	1	.051	1.481	.998	2.198
	Self-care ability			1.284	2	.526			
	Partially self-care	−.064	.653	.009	1	.922	.938	.261	3.373
	Fully self-care	−.316	.661	.228	1	.633	.729	.200	2.663
	Whether sedentary(1)	.393	.223	3.107	1	.078	1.482	.957	2.295
	BMI			5.473	3	.140			
	Normal	−.423	.314	1.818	1	.178	.655	.354	1.212
	Overweight	−1.029	.476	4.675	1	.031	.358	.141	.908
	Obesity	−629	.355	3.138	1	.076	.533	.266	1.069
	Defecation time			20.621	3	.000			
	Morning	−.851	.200	18.158	1	.000	.427	.289	.632
	Medium	.721	.963	.561	1	.454	2.057	.312	13.579
	Evening	−1.027	.556	3.415	1	.065	.358	.121	1.064
	Whether spicy diet			10.891	4	.028			
	2 degrees	.603	.271	4.935	1	.026	1.827	1.074	3.111
	3 degrees	−.984	.606	2.637	1	.104	.374	.114	1.226
	4 degrees	1.024	1.207	.719	1	.396	2.783	.261	29.650
	5 degrees	−.300	.297	1.022	1	.312	.741	.414	1.325
	Constants	.406	1.223	.110	1	.740	1.501		

* Variables entered in step 1: age, family history of constipation, education level, self-care ability, sedentary or not, BMI, bowel movement time per day, spicy diet or not.

### 3.4. Conclusion

For hospitalized elderly people, regular morning defecation is more helpful to slow the occurrence of functional constipation. At the same time, not eating spicy foods can also help slow the occurrence of functional constipation. This helps us in clinical education for patients with functional constipation or potential functional constipation, we can advise them to eat less spicy food and help them to defecate regularly as much as possible.

## 4. Discussion

The results of this study showed that the prevalence of FC among elderly inpatients in our hospital was 50.1%, which was significantly higher than the prevalence reported in domestic and international studies. The possible reasons for this are: in the general research literature, the subjects are mostly elderly people in a certain area, but the subjects of this study are inpatients, so there are their special characteristics. It also indicates that the prevalence of FC in elderly people in Yunnan is higher, so it should be more concerned.

The present investigation found that elderly people with irregular bowel movement time and spicy food 1 to 2 times a week had a high prevalence of FC, while family history of constipation, literacy, sedentary, self-care ability, age, and BMI had some influence on FC. The author reviewed the relevant literature and found that family history of constipation is a high risk factor for the occurrence of FC, and patients with a family history of constipation in their parents or other immediate family members are 1.589 times more likely to have FC than those without a family history of constipation,^[7]^ which may be related to genetic susceptibility and similar living environment.

As the elderly age, organ function decreases, chewing ability decreases, digestive juices secretion decreases, eating volume decreases, and diet is mainly fine food intake, dietary fiber is insufficient, resulting in prolonged colonic transmission time, decreased stool volume, less intestinal stimulation, and patients’ bowel movement is not obvious.^[4]^ At the same time, the older the patient is, the weaker the contraction of abdominal wall muscles, levator muscles and intestinal smooth muscles, leading to decreased bowel function^[8]^; in addition, elderly patients often take more medications due to the coexistence of multiple diseases, and some diseases and medications tend to cause constipation, so the age factor is an important reason for the occurrence of FC in the elderly. Irregular bowel timing is an independent risk factor for FC, and in the present investigation, the proportion of FC occurring with regular morning bowel movements was low. The rise reflex in the morning can promote the peristalsis of the colon and facilitate defecation.^[4]^ If patients do not develop the conditioned reflex to defecate regularly, it can lead to constipation caused by relaxation of intestinal muscles.^[9,10]^ Irregular defecation time is a variable factor, and the occurrence of constipation can be reduced by changing defecation habits and defecating regularly through health education for elderly FC patients.

Different dietary habits lead to variability in the prevalence of FC, and the present study suggests that the prevalence of FC is higher in those who prefer spicy foods than in those who eat a completely light diet. This finding is consistent with most of the reports at home and abroad,^[11,12]^ and the author believes that long-term consumption of spicy food will stimulate the intestinal tract,^[13]^ which will raise the threshold of the intestinal nervous system, thus slowing down the gastrointestinal motility, reducing the secretion of digestive juices, reducing intestinal contraction and peristalsis, and thus constipation; at the same time, long-term preference for spicy and stimulating food will also have certain effects on the intestinal flora and its microenvironment, making it more likely to develop constipation.

The prevalence of FC in elderly inpatients in Yunnan is high and the influencing factors are complex, so targeted prevention and treatment should be carried out to guide the elderly group to correctly understand FC. We can educate elderly hospitalized patients with functional constipation, encourage them to train to defecate as regularly as possible, and pay attention to reducing the intake of spicy food.

This study limitation is that we did not have a complete collection of medication histories of hospitalized patients. We only paid attention to the influence of these patients’ underlying diseases and the main diseases of this visit on functional constipation, and ignored the influence of whether they had multiple drugs on functional constipation.

## Author contributions

**Data curation:** Xiaobin Wang.

**Formal analysis:** Xuejiao Xian, Xiaobin Wang.

**Investigation:** Jie Liu.

**Methodology:** Xiaobin Wang.

**Resources:** Jie Liu.

**Supervision:** Hongjun Yang.

**Validation:** Jie Liu.

**Visualization:** Hongjun Yang.

**Writing – original draft:** Xuejiao Xian.

**Writing – review & editing:** Hongjun Yang.
